# Rapid Microarray-Based Detection of Rifampin, Isoniazid, and Fluoroquinolone Resistance in Mycobacterium tuberculosis by Use of a Single Cartridge

**DOI:** 10.1128/JCM.01249-17

**Published:** 2018-01-24

**Authors:** Juliane Havlicek, Beatrice Dachsel, Peter Slickers, Sönke Andres, Patrick Beckert, Silke Feuerriegel, Stefan Niemann, Matthias Merker, Ines Labugger

**Affiliations:** a Alere Technologies GmbH, Jena, Germany; b National Reference Center for Mycobacteria, Research Center Borstel, Borstel, Germany; c Molecular and Experimental Mycobacteriology, Research Center Borstel, Borstel, Germany; d German Center for Infection Research, Partner Site Hamburg-Lübeck-Borstel, Borstel, Germany; Carter BloodCare & Baylor University Medical Center

**Keywords:** Mycobacterium tuberculosis, diagnostics

## Abstract

The rapid and robust identification of mutations in Mycobacterium tuberculosis complex (MTBC) strains mediating multidrug-resistant (MDR) and extensively drug-resistant (XDR) phenotypes is crucial to combating the MDR tuberculosis (TB) epidemic. Currently available molecular anti-TB drug susceptibility tests either are restricted to a single target or drug (i.e., the Xpert MTB/RIF test) or present a risk of cross-contamination due to the design limitations of the open platform (i.e., line probe assays). With a good understanding of the technical and commercial boundaries, we designed a test cartridge based on an oligonucleotide array into which dried reagents are introduced and which has the ability to identify MTBC strains resistant to isoniazid, rifampin, and the fluoroquinolones. The melting curve assay interrogates 43 different mutations in the rifampin resistance-determining region (RRDR) of *rpoB*, *rpoB* codon 572, *katG* codon 315, the *inhA* promoter region, and the quinolone resistance-determining region (QRDR) of *gyrA* in a closed cartridge system within 90 min. Assay performance was evaluated with 265 clinical MTBC isolates, including MDR/XDR, non-MDR, and fully susceptible isolates, from a drug resistance survey performed in Swaziland in 2009 and 2010. In 99.5% of the cases, the results were consistent with data previously acquired utilizing Sanger sequencing. The assay, which uses a closed cartridge system in combination with a battery-powered Alere q analyzer and which has the potential to extend the current gene target panel, could serve as a rapid and robust point-of-care test in settings lacking a comprehensive molecular laboratory infrastructure to differentiate TB patients infected with MDR and non-MDR strains and to assist clinicians with their early treatment decisions.

## INTRODUCTION

With more than 10 million new cases of Mycobacterium tuberculosis infection occurring every year and an estimated 1.8 million deaths from tuberculosis (TB) occurring in 2015, TB remains one of the central health problems worldwide. One major cause of the global TB situation and the challenges associated with disease elimination is the increasing occurrence of M. tuberculosis complex (MTBC) bacteria resistant to antibiotics. In 2015 alone there were an estimated 480,000 new cases of multidrug-resistant TB (MDR-TB) and approximately 250,000 deaths from MDR-TB, defined as resistance to rifampin and isoniazid. The average proportion of MDR-TB cases with additional resistance to at least one fluoroquinolone and a second-line injectable agent (extensively drug-resistant TB [XDR-TB]) was 9.5%, similar to estimates in previous years (1, 2). The increasing occurrence of drug-resistant MTBC strains is associated, inter alia, with a diagnostic gap in many regions with a high incidence of TB (1). It is projected that MDR-TB is detected in only one out of three MDR-TB patients worldwide, and the rates of treatment success for MDR-TB patients are dramatically low at 48% (1). A successful treatment outcome is essentially associated with the early administration of adequate drugs and, therefore, with individualized drug susceptibility testing (3–5).

Culture-based methods are still considered the “gold standard” for the diagnosis of infection with MTBC bacteria and the detection of resistance in MTBC strains, but these methods do not enable early treatment decisions to be made. Additionally, these methods are heavily burdened with the requirement for a complex laboratory infrastructure (6, 7). Molecular tests subsequently become additionally important because the molecular mechanisms of MTBC drug resistance are well characterized. These mechanisms include, e.g., mutations in defined regions of the *rpoB* gene, coding for an RNA polymerase subunit; *katG*, coding for a catalase; the *inhA* promoter region, controlling RNA transcripts for an enoyl-acyl carrier protein involved in fatty acid biosynthesis; and *gyrA*, coding for a subunit of the DNA gyrase. Approximately 97% of rifampin-resistant MTBC isolates harbor mutations in the 81-bp rifampin resistance-determining region (RRDR) of the *rpoB* gene (codons 507 to 533), wherein the most frequent mutations affect codons 516, 526, and 531 (Escherichia coli numbering) (8–10). Several studies showed that mutations at amino acid position 572 in the *rpoB* gene also contribute to rifampin resistance in MTBC strains (11–13) and are not interrogated by currently available molecular drug susceptibility tests (DSTs). Mutations in codon 315 of the *katG* gene or in the *inhA* promoter region (positions −8 and −15) are associated with isoniazid resistance in 70 to 90% of isoniazid-resistant MTBC strains (8–10). Mutations in the quinolone resistance-determining region (QRDR) of *gyrA* (codons 88 to 94) are associated with resistance to fluoroquinolones, the backbone of MDR-TB treatment, in more than 80% of MTBC clinical isolates (10, 14, 15).

Over the past decade, molecular tests (e.g., line probe assays or the Xpert MTB/RIF test) have been developed to identify resistance-related mutations (16). However, these tests are restricted by either their ability to investigate only a single resistance target or the risk of contamination that they present due to the use of an open platform. In this report, we describe a rapid, sensitive, and simple test which is performed with a single prototype cartridge and the Alere q analyzer. The original functional use of the Alere q platform was for the detection of HIV using competitive reporter-monitored amplification (CMA), in which Cy5-labeled reporter oligonucleotides are competitively hybridized onto a probe array (17). A new method based on melting curve analysis has now been implemented. The special feature of the assay described here is that the fluorescence measurement is performed on the solid phase. On this account, just one fluorescent dye is needed for analysis, in contrast to other melting curve assays described previously (18–22). This enables the simultaneous detection of many different mutations which are associated with resistance to rifampin, isoniazid, and fluoroquinolones.

## MATERIALS AND METHODS

### Strains of mycobacteria.

The MTBC strains used for the clinical studies (see Table S1 in the supplemental material) were isolated under standard routine conditions at the National Reference Center for Mycobacteria, Research Center Borstel, Borstel, Germany. The nontuberculous mycobacteria (NTM) tested were incubated in tubes with Middlebrook and Cohn 7H10 agar (BD Diagnostics, Franklin Lakes, NJ, USA) at 37°C for multiple days at Alere Technologies GmbH, Jena, Germany. To obtain inactivated cell culture material, the strains were treated with ultrasound and heated at 99°C for 15 min each.

For the clinical evaluation, we tested 265 clinical MTBC isolates which were previously analyzed by Sanger sequencing of the respective resistance-associated gene regions. The isolates were derived from a national survey of drug resistance in Swaziland, a region with a high incidence of MDR-TB (23). The selection covered MDR-TB strains (*n* = 116), XDR-TB strains (*n* = 1), non-MDR strains (*n* = 19), and a random selection of fully susceptible strains (*n* = 129).

### Isolation of genomic DNA.

For isolation of bacterial genomic DNA, inactivated culture material (from Lowenstein-Jensen slants) was resuspended in 1 ml TET buffer (Tris, ETDA, Tween 20) and filtered through a 5-μm-pore-size filter (Omnifix Braun, Melsungen, Germany) to obtain single cells. The bacterial solution was washed twice with TET buffer, centrifuged at 5,000 × *g* for 5 min, and, finally, adjusted to an optical density of 0.01 in TET buffer. Subsequently, 400 μl of the bacterial solution was disrupted using a bead beating device (VWR, Erlangen, Germany) and 300 mg of glass beads (<106 μm; Sigma-Aldrich, St. Louis, MO, USA), which were centrifuged at 5,000 × *g* for 80 s. The cell lysate obtained was centrifuged at 5,000 × *g* for 10 s, and the supernatant was filtered through a 10-μm-pore-size filter (Mobitec, Eupen, Belgium) and centrifuged at 5,000 × *g* for 1 min. The genomic DNA was extracted from 100 μl of this cell lysate using a QIAamp blood minikit (Qiagen, Hilden, Germany) according to the manufacturer's instructions. DNA quantification was performed using a Quant-iT PicoGreen double-stranded DNA kit (Invitrogen, Carlsbad, CA, USA). Finally, the DNA sequences of the MBTC strains were determined by GATC Biotech AG (Constance, Germany) and analyzed using GENtle software (http://gentle.magnusmanske.de).

### Primers, TaqMan probes, and array probes.

Sequence information for all primers, array probes, and TaqMan probes is available in Table S3. The array probes had a C-7 amino linker for immobilization on the array surface. Forward primers for melting curve analysis were labeled with Cy5 at the 5′ end; the reverse primers used for melting curve analysis as well as the primers used for TaqMan analysis were unlabeled. All oligonucleotides were synthesized by Eurogentec, Cologne, Germany.

### Efficiency of multiplex target amplification using real-time PCR.

Real-time PCR was performed with primers and fluorescence-labeled TaqMan probes specific for the targets. Due to the limitation in the fluorescence channels of the 7500 real-time PCR system used (Applied Biosystems, Foster City, CA, USA), the multiplex amplification was analyzed in two reactions. Sequence-specific primers were added iton both reaction mixtures, whereas the respective TaqMan probes were added to one or the other reaction mixture. MTBC strains are characterized by a high GC content, which required an adaptation of the PCR buffer used (24). To improve the PCR amplification in connection with an increased specificity and reaction yield, betaine and tetramethylammonium chloride were added to the reaction mixture (25–27). Therefore, the amplification was performed in a final reaction volume of 100 μl containing TET buffer (Tris, ETDA, Tween 20), 75 mM Tris-HCl (pH 8.5; Alere Technologies GmbH, Jena, Germany), 3 mM magnesium chloride (Sigma-Aldrich, St. Louis, MO, USA), one betaine tablet (resulting in 2 M betaine; Friedrich Alexander University, Erlangen, Germany), 100 mM tetramethylammonium chloride (Alere Technologies GmbH, Jena, Germany), 0.2 mM deoxynucleoside triphosphates (Thermo Fisher Scientific, Waltham, MA, USA), 0.6 μM each forward primer (Table S3), 0.2 μM each reverse primer (Table S3), 0.2 μM the corresponding TaqMan probes (Table S3), 12.5 U of BTR Hot Start *Taq* (biotechrabbit, Henningsdorf, Germany), genomic DNA from the M. tuberculosis H37Rv reference strain (10^2^ to 10^4^ copies per reaction mixture; Tebu-Bio, Offenbach, Germany), and 10^4^ copies per reaction mixture of the internal process control (Ionian Technologies, San Diego, CA, USA). For the negative controls, the same reaction mix but without any template was used. The reaction was performed using a model 7500 real-time PCR system with the following conditions: 95°C for 2 min following 40 cycles at 95°C for 10 s, 64°C for 30 s, and 72°C for 30 s. The fluorescence values obtained were used to determine a standard calibration curve as well as to calculate the efficiency of the multiplex amplification of the MTBC-specific targets.

### Melting curve analysis for detection of MTBC drug resistance.

**FIG 1:**
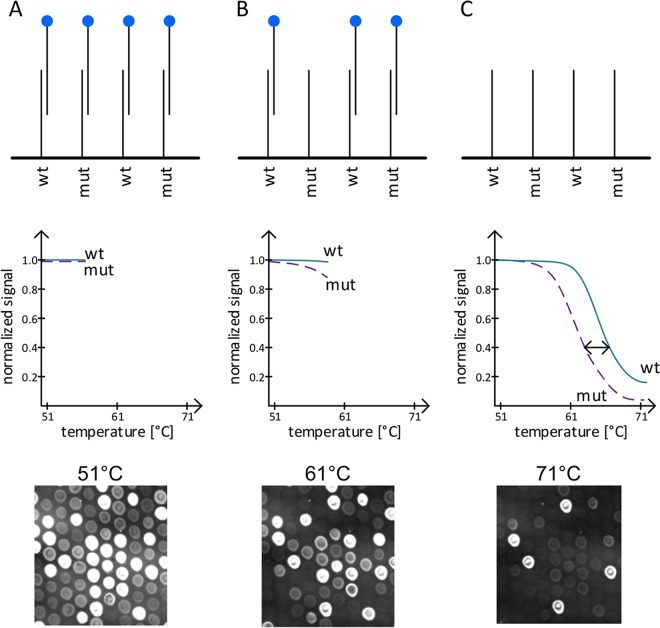
Principle of isolate detection by the melting curve assay. For the drug resistance-associated targets *rpoB* RRDR, *rpoB* codon 572, *katG*, *gyrA* QRDR, and the *inhA* promoter region, probes representing the wild-type (wt) and mutant (mut) genotypes were spotted onto the array. (A) Cy5-labeled amplicons (e.g., those for the wild type) are generated during the multiplex amplification and initially bind to the specific sequence of the wild type as well as the specific sequences of the unspecific mutant probes due to the low temperature profile. (B) With an increase in temperature, the unspecific amplicon bond dissolves, resulting in a loss of the signal at the nonmatching mutant probes but retention of the signal at the matching wild-type probes. (C) At the final temperature of 71°C, a very low fluorescence signal is detected on the array spots, as almost all bonds are dissolved.

The melting curve assay was performed in a prototype cartridge (Alere Technologies GmbH, Jena, Germany) on the Alere q analyzer. After the reaction mix was applied to the cartridge, all steps from amplification to detection were performed automatically. In the first step, a multiplex amplification of the defined targets was performed using Cy5-labeled forward primers. The fluorescence-labeled amplicons were denatured, and the single strands were hybridized to the probes carrying either the wild-type or the mutant genotype sequence spotted on the array (Fig. 1A). At the beginning of the melting curve profile, nonmatching amplicon-probe bonds were dissolved (Fig. 1B), and with a further increase in temperature, the amplicons complementary to the probe sequence at their specific melting points were also dissolved (Fig. 1C).

Our assay used 61 immobilized probes representing the wild-type or mutant genotypes for the detection of 43 different mutations mediating resistance to rifampin, isoniazid, and fluoroquinolones. With the melting curve assay, single nucleotide polymorphisms (SNPs) could be precisely identified in codons 511 [Leu511Pro, C(T/C)G], 513 [Ser513Leu, C(A/T)A; Ser513Lys, (C/A)AA; Ser513Pro, C(A/C)A], 516 [Asp516Phe, (G/T)(A/T)C; Asp516Val, G(A/T)C; Asp516Tyr, (G/T)AC], 518 [Asn518Ser, A(A/G)C], 522 [Ser522Gln, (T/C)(C/A)G; Ser522Leu, T(C/T)G; Ser522Trp, T(C/G)G], 526 [His526Asp, (C/G)AC; His526Arg, C(A/G)C; His526Asn, (C/A)AC; His526Leu, C(A/T)C; His526Tyr, (C/T)AC; His526Cys, (C/T)(A/G)C; His526Gln, CA(C/A); His526Pro, C(A/C)C; His526Ser, (C/A)(A/G)C], 531 [Ser531Leu, T(C/T)G; Ser531Trp, T(C/G)G], 533 [Leu533Pro, C(T/C)G], and 572 [Ile572Phe, (A/T)TC] (28–38). For the detection of isoniazid resistance, probes carrying SNPs in *katG* codon 315 [Ser315Asn, A(G/A)C; Ser315Ile, A(G/T)C; Ser315Gly, (A/G)GC; Ser315Thr, A(G/C)C; Ser315Thr, A(G/C)(C/A)] and within the *inhA* promoter region at positions −8 (−8T → A, −8T → C) and −15 (−15C → T) were applied (39–44). Resistance to fluoroquinolones is mostly associated with mutations in the *gyrA* target region (10, 14, 15). Therefore, specific probes carrying SNPs in codons 88 [Gly88Cys, (G/T)GC], 89 [Asp89Asn, (G/A)AC], 90 [Ala90Val, G(C/T)G], 91 [Ser91Pro, (T/C)CG], and 94 [Asp94Ala, G(A/C)C; Asp94Asn, (G/A)AC; Asp94Gly, G(A/G)C; Asp94His, (G/C)AC; Asp94Tyr, (G/T)AC; Asp94Val, G(A/T)C] were spotted onto the array (45–48). In addition, a specific probe carrying an SNP at position 95 defining the phylogenetic Ser95Thr mutation [A(G/C)C], which is not associated with resistance to fluoroquinolones (49), was also implemented.

The melting curve assay was performed in a reaction volume of 100 μl comprising 75 mM Tris hydrochloride, pH 8.5 (Alere Technologies GmbH, Jena, Germany), 3 mM magnesium chloride (Sigma-Aldrich, St. Louis, MO, USA), 1 betaine tablet (resulting in 2 M betaine; Friedrich Alexander University, Erlangen, Germany), 100 mM tetramethylammonium chloride (Alere Technologies GmbH, Jena, Germany), 0.2 mM a deoxynucleoside triphosphate mixture (Thermo Fisher Scientific, Waltham, MA, USA), 0.6 μM each Cy5-labeled forward primer (Table S3), 0.2 μM each reverse primer (Table S3), 25 U of BTR Hot Start *Taq* (biotechrabbit, Henningsdorf, Germany), 10^4^ copies per reaction mixture of the internal process control (Ionian Technologies, San Diego, CA USA), and 1 μl of genomic MTBC DNA or crude cell culture material. The genomic DNAs with known genotypes were tested with 3-fold of the concentration at the limit of detection. The extracted bacterial DNA from the patient-derived isolates in the clinical validation study as well as the crude cell lysate was diluted 1:1,000 with Tris-EDTA buffer (Sigma-Aldrich, St. Louis, MO, USA). Negative-control reactions were also performed. Amplification was performed at 95°C for 2 min following 40 cycles at 95°C for 10 s, 64°C for 30 s, and 72°C for 30 s. A magnesium chloride pellet stored in the cartridge (resulting in 200 mM magnesium chloride; Alere Technologies GmbH, Jena, Germany) was dissolved in the reaction mix containing the generated Cy5-labeled amplicons. After denaturation at 95°C for 2 min, the amplicons were hybridized on the array probes at 30°C for 2 min. Subsequently, a washing step using buffer WB2.10 (Alere Technologies GmbH, Jena, Germany) and a magnesium chloride pellet (resulting in 200 mM magnesium chloride; Alere Technologies GmbH, Jena, Germany) was performed. Finally, an image of the array was acquired during the melting curve analysis over a temperature range of from 51°C to 71°C in 2°C increments.

### Data processing and analysis.

The image series acquired was analyzed using Iconoclust software (Alere Technologies GmbH, Jena, Germany). The array spots were identified using a defined grid. The raw fluorescence signal of each array spot was calculated by subtraction of the nonspecific background from its absolute value. Afterwards an interpolation was performed to receive a continuous signal. If the raw signal exceeded 0.05, 0.1, or 0.2 (depending on the probe being considered), it was declared to be detected and the signal was normalized. For each probe, the temperature at a signal level of 0.4 was determined. For corresponding probe pairs representing wild-type and mutant genotypes, the ratio of the temperatures was calculated and was considered the so-called discrimination factor. A discrimination factor of <1 represented the wild-type genotype, and a discrimination factor of >1 represented the mutant genotype.

## RESULTS

Our melting curve assay comprised two parts: amplification of the selected target regions (the *rpoB* RRDR, *rpoB* codon 572, *katG* codon 315, the *gyrA* QRDR, and the *inhA* promoter region) by a multiplex PCR and detection of mutations by a melting curve analysis. The efficiency of the multiplex amplification was determined by use of a standard curve (50). It was calculated to be excellent for all targets, as judged by efficiencies of ≥0.97 (data not shown). Melting curve analysis was done with a DNA array carrying several immobilized hybridization probes. Initially, several different variants of the probe sequences were screened to find pairs which yielded a reliable discrimination between wild-type and mutant strains (data not shown).

### Sensitivity and specificity.

**FIG 2:**
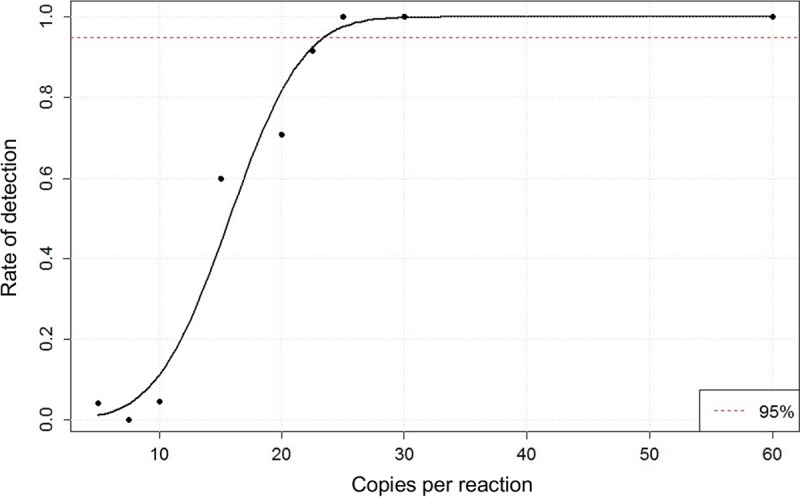
Sensitivity of the melting curve assay for the multiplex amplification of the MTBC strain-specific targets *rpoB* RRDR, *rpoB* codon 572, katG, *gyrA* QRDR, and the *inhA* promoter region using the prototype cartridge and the Alere q analyzer. The sensitivity was determined to be 24 copies per reaction mixture with a 95% confidence interval of 21 to 27 copies per reaction mixture. For each concentration (number of copies per reaction mixture), 25 tests were performed. The dashed line shows the threshold where in 95% of the cases MTBC DNA could be detected.

The sensitivity was determined using genomic DNA from M. tuberculosis reference strain H37Rv over a concentration range of 5 to 60 copies per reaction mixture. An increase in the detection rate was observed with increasing DNA concentrations. The detection limit of the melting curve assay was 24 copies per reaction mixture (95% confidence interval, 21 to 27 copies per reaction mixture) (Fig. 2).

**FIG 3:**
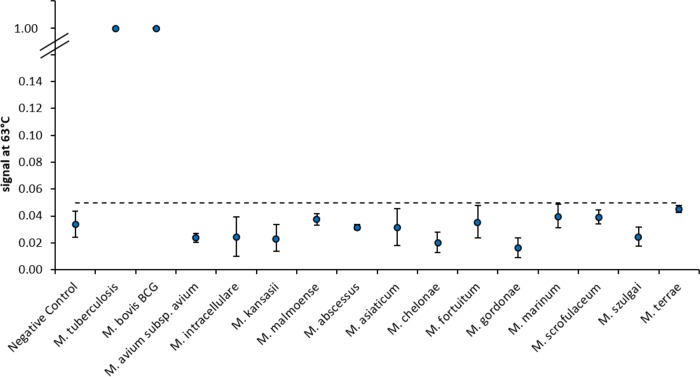
Specificity of the melting curve assay. The *x* axis shows the mycobacterial species tested, including M. tuberculosis, M. bovis BCG, and nontuberculous mycobacteria. A probe specific for MTBC strains was used as an internal process control for verification of species specificity. Hybridization signals of >0.05 at 63°C are defined as positive and indicate the presence of strains belonging to the MTBC; thus, M. tuberculosis and M. bovis BCG were measured to be positive at the specific spot. NTM showed no cross-reaction with 106 copies per reaction in a reaction mix (hybridization signal < 0.05).

In addition to probes for the resistance targets, a probe for the highly specific detection of MTBC strains was applied. DNA isolated from different nontuberculous mycobacteria, such as M. intracellulare, M. malmoense, M. asiaticum, M. fortuitum, and M. marinum, was tested. No unspecific reaction could be observed. On the contrary, other species of the MTBC, like M. bovis BCG, showed signals comparable to those produced by M. tuberculosis (Fig. 3).

### Detection of mutations in *rpoB*, *katG*, *gyrA*, and the *inhA* promoter region.

A set of 30 different MTBC isolates whose sequences were verified were analyzed. Overall, these isolates carried 18 mutations in the *rpoB* RRDR, 1 mutation in *rpoB* codon 572, 5 different SNPs in *katG* codon 315, 9 mutations in the *gyrA* QRDR, and 2 SNPs in the *inhA* promoter region. The results for all isolates are summarized in Table 1. When genomic wild-type DNA was tested, a discrimination factor (i.e., the ratio of the temperature for the wild type and the mutant probes at a signal level of 0.4) of <1 was observed with all probes, whereas a mutation within the respective target region could be identified by determination of a discrimination factor of >1 with the corresponding probe. In addition, there were also discrimination factors of >1 for the noncorresponding probes (Table 1); however, the mutation could be correctly identified, as the highest value of the discrimination factor with the matching probe was always measured. With the exception of the *rpoB* Leu511Arg mutation in isolate 853/07 and the *rpoB* Ile569Val mutation in isolate 10523/05, all mutations were correctly identified.

**TABLE 1:** Discrimination factors determined with array probes^*a*^

Isolate	Discrimination factor obtained for the following mutations with the indicated probes:																						
	*rpoB* RRDR																						
	511Pro_v02	513Leu_v03	513Lys_v02	513Pro_v04	516Tyr_v03	516Val_v03	516Phe_v03	518Ser_v02	522Gln_v03	522Leu_v02	522Trp_v01	526Asp_v04	526Arg_v04	526Asn_v06	526Leu_v04	526Tyr_v04	526Cys_v03	526Gln_v03	526Pro_v04	526Ser_v01	531Leu_v04	531Trp_v05	533Pro_v04
H37Rv	0.93	0.94	0.90	0.95	0.96	0.93	0.93	0.89	0.84	0.88	0.87	0.88	0.89	0.89	0.94	0.93	0.88	0.90	0.96	0.89	0.93	0.89	*
3736/04	**1.13**	0.94	0.87	0.92	0.96	0.94	0.94	0.89	0.84	0.87	0.88	0.93	0.93	0.93	0.97	0.96	0.91	0.94	0.95	0.93	0.94	0.99	*
698/05	0.87	**1.01**	0.94	**1.09**	0.98	0.96	0.94	0.90	0.83	0.87	0.87	0.89	0.90	0.90	0.95	0.94	0.90	0.93	0.97	0.89	0.91	0.89	*
10502/06	0.89	**1.04**	0.93	**1.01**	0.97	0.96	0.94	0.89	0.83	0.86	0.86	0.88	0.89	0.89	0.94	0.92	0.86	0.91	0.95	0.92	0.94	0.91	*
6695/04	0.92	0.92	0.92	0.95	0.96	0.93	0.91	**1.05**	0.85	0.88	0.92	0.89	0.88	0.91	0.93	0.92	0.86	0.90	0.96	0.87	**	**	**1.06**
7941/01	0.92	0.94	0.91	0.95	0.96	0.94	0.93	0.90	0.92	**1.09**	0.97	0.88	0.90	0.87	0.94	0.92	0.85	0.90	0.95	0.88	0.94	0.93	*
49-02-SR4a	0.93	0.94	0.91	0.95	0.95	0.94	0.93	0.89	0.98	**1.04**	**1.15**	0.86	0.88	0.84	0.92	0.90	0.85	0.89	0.93	0.86	0.93	0.89	*
5976/01	0.93	0.94	0.91	0.95	0.96	0.93	0.93	0.89	0.83	0.87	0.86	0.93	0.97	0.93	**1.10**	**1.01**	0.99	0.97	**1.04**	0.96	0.94	0.95	*
4787/03	0.93	0.94	0.91	0.96	0.96	0.94	0.93	0.89	0.93	0.85	0.87	0.95	0.97	0.97	0.99	**1.07**	**1.00**	0.91	**1.05**	0.94	0.92	0.88	*
3307/03	0.93	0.93	0.90	0.94	0.96	0.94	0.93	0.88	0.88	0.85	0.86	0.98	0.92	**1.08**	0.98	**1.03**	0.95	0.96	0.99	0.97	0.94	0.89	*
10427/01	0.93	0.95	0.91	0.96	0.96	0.94	0.92	0.89	0.89	0.84	0.85	0.98	**1.04**	0.98	**1.00**	**1.07**	**1.14**	0.94	0.98	**1.05**	0.91	0.91	*
2822/06	0.93	0.93	0.91	0.95	0.96	0.94	0.93	0.89	0.86	0.85	0.86	**1.15**	0.96	**1.05**	0.98	**1.07**	0.97	0.93	0.97	0.97	0.92	0.89	*
H37Rv-SR4k	0.93	0.94	0.91	0.95	0.96	0.93	0.93	0.89	0.84	0.86	0.89	0.93	0.99	0.97	**1.04**	**1.07**	0.94	**1.05**	**1.12**	0.92	***	***	*
4724/03	0.90	0.94	0.91	0.95	0.96	0.94	0.93	0.90	0.94	0.87	0.88	0.95	**1.04**	0.93	**1.00**	**1.02**	**1.01**	0.96	**1.02**	0.93	0.98	0.98	*
368/01	0.92	0.94	0.91	0.95	0.96	0.94	0.93	0.89	0.86	0.87	0.87	0.90	0.88	0.91	0.94	0.93	0.87	0.90	0.96	0.89	**1.07**	**1.01**	*
H37Rv-SR8a2	0.93	0.94	0.91	0.95	0.96	0.94	0.93	0.89	0.84	0.87	0.90	0.88	0.92	0.90	0.95	0.93	0.87	0.90	0.96	0.87	***	***	*
4709/09	0.91	0.94	0.91	0.95	0.97	0.94	0.93	0.90	0.86	0.87	0.90	0.91	0.91	0.91	***	0.96	0.89	0.91	0.99	0.87	0.94	***	*
12401/03	0.90	0.94	0.91	0.95	0.96	0.94	0.93	0.89	0.88	0.88	0.87	0.88	0.90	0.91	0.95	0.93	0.92	0.90	0.97	0.89	**1.05**	**1.01**	*
3355/02	0.93	0.94	0.91	0.95	0.96	0.94	0.93	0.89	0.83	0.87	0.88	0.90	0.90	0.90	0.95	0.94	0.88	0.94	0.97	0.89	***	0.90	*
3429/03	0.92	0.94	0.91	0.96	0.96	0.94	0.93	0.89	0.84	0.87	0.87	0.89	0.90	0.90	0.95	0.93	0.88	0.91	0.97	0.90	0.97	0.92	*
1429/02	0.93	0.94	0.91	0.97	0.96	0.94	0.93	0.88	0.84	0.87	0.91	0.90	0.93	0.90	0.95	0.93	0.90	0.94	0.98	0.88	0.97	0.98	*
8085/03	0.93	0.91	0.88	0.96	0.99	**1.07**	**1.03**	0.85	0.83	0.86	0.86	0.90	0.89	0.89	0.94	0.93	0.89	0.91	0.95	0.88	0.94	0.89	*
853/07	0.97	0.94	0.96	0.97	**1.08**	**1.00**	**1.04**	0.84	0.82	0.86	0.84	0.90	0.89	0.89	0.94	0.94	0.88	0.90	0.95	0.87	0.96	0.90	*
H37Rv-SO4a	0.92	0.94	0.91	0.95	0.96	0.93	0.93	0.89	0.84	0.86	0.87	0.91	0.92	0.92	0.95	0.94	0.93	0.93	0.98	0.93	0.96	0.93	*
4535/04	0.90	0.94	0.90	0.95	0.96	0.95	0.93	0.89	0.83	0.87	0.86	0.88	0.88	0.90	0.94	0.93	0.87	0.89	0.96	0.90	**1.07**	0.93	*
464/11	0.93	0.94	0.91	0.95	0.96	0.94	0.93	0.88	0.83	0.87	0.87	0.89	0.89	0.89	0.94	0.92	0.87	0.91	0.96	0.87	**1.07**	0.97	*
1598/06	0.92	0.94	0.90	0.95	0.96	0.93	0.93	0.88	0.86	0.87	0.88	0.89	0.89	0.90	0.94	0.92	0.89	0.91	0.96	0.92	**1.05**	0.95	*
10523/05	0.93	0.93	0.90	0.95	0.96	0.94	0.93	0.88	0.84	0.87	0.87	0.89	0.88	0.90	0.94	0.92	0.86	0.90	0.95	0.87	**1.08**	0.98	*
3075/05	0.93	0.94	0.90	0.95	0.96	0.93	0.92	0.89	0.83	0.87	0.87	0.88	0.88	0.89	0.94	0.92	0.86	0.90	0.95	0.87	**1.08**	0.96	*
8444/05	0.90	0.94	0.91	0.95	0.96	0.94	0.93	0.88	0.87	0.87	0.87	0.89	0.93	0.91	0.94	0.94	0.88	0.94	0.97	0.89	**1.06**	0.92	*

Isolate	Discrimination factor obtained for the following mutations with the indicated probes:																						
	*rpoB*, 572Phe_v04	*katG*					*inhA*			*gyrA* QRDR											Controls		
		315Asn_v06	315Ile_v01	315Thr1_v01	315Thr2_v04	315Gly_v04	−8T>A_v02	−8T>C_v02	−15C>T_v02	88Cys_v01	89Asn_v04	90Val_v01	91Pro_v04	94Ala_v01	94Asn_v02	94Gly_v02	94His_v04	94Tyr_v02	94Val_v02	95Thr_v01	M. smegmatis	M. tuberculosis	sp_02
H37Rv	0.97	0.96	0.96	0.93	0.92	0.94	0.96	0.95	0.96	0.93	0.98	0.94	0.96	0.98	0.99	0.93	0.98	0.98	0.97	0.99	x	x	−
3736/04	0.97	0.96	0.96	0.92	0.91	0.94	0.98	0.96	0.96	0.91	0.99	0.94	0.97	**1.01**	0.98	0.98	0.99	0.97	0.95	**1.02**	x	x	−
698/05	0.97	**1.05**	**1.04**	**1.11**	**1.07**	**1.00**	0.96	0.92	0.92	0.93	0.99	0.93	0.97	0.98	0.97	0.95	0.98	0.97	0.95	**1.02**	x	x	−
10502/06	0.97	**1.05**	**1.03**	**1.11**	**1.05**	**0.99**	0.98	0.96	0.95	0.93	1.00	0.95	0.98	0.98	0.98	0.94	0.98	0.97	0.95	**1.01**	x	x	−
6695/04	0.98	**1.06**	**1.04**	**1.11**	**1.07**	**1.00**	0.94	0.98	0.97	0.93	0.94	0.93	0.97	0.97	0.98	0.94	0.98	0.97	0.95	**1.01**	x	x	−
7941/01	0.97	0.96	0.96	0.92	0.92	0.94	0.94	0.92	0.90	0.90	0.97	0.89	0.93	0.96	0.96	0.95	0.98	0.96	0.94	**1.06**	x	x	−
49-02-SR4a	0.97	0.95	0.96	0.91	0.91	0.94	0.99	0.97	0.96	0.94	0.99	0.96	0.98	0.99	0.98	0.94	0.99	0.97	0.95	**1.01**	x	x	−
5976/01	0.96	**1.06**	**1.04**	**1.12**	**1.07**	**1.00**	0.95	0.93	0.92	0.91	0.98	0.90	0.94	0.98	0.97	0.96	0.98	0.97	0.96	**1.03**	x	x	−
4787/03	0.96	**1.05**	**1.04**	**1.11**	**1.07**	**1.00**	0.93	0.93	0.91	0.93	0.98	0.93	0.97	0.99	0.98	0.93	0.98	0.97	0.95	**1.02**	x	x	−
3307/03	0.97	0.96	0.96	0.92	0.91	0.94	0.91	0.88	**1.06**	0.90	0.97	0.89	0.93	0.97	0.97	0.93	0.98	0.96	0.95	**1.05**	x	x	−
10427/01	0.97	**1.06**	**1.04**	**1.12**	**1.07**	**1.00**	0.94	0.92	0.90	0.90	0.97	0.90	0.93	0.97	0.97	0.93	0.98	0.96	0.94	**1.05**	x	x	−
2822/06	0.97	0.95	0.96	0.92	0.91	0.94	0.89	0.87	**1.07**	0.90	0.97	0.88	0.93	0.97	0.96	0.95	0.97	0.96	0.94	**1.08**	x	x	−
H37Rv-SR4k	0.96	0.95	0.96	0.92	0.91	0.94	0.93	0.91	0.90	0.90	0.97	0.89	0.93	0.97	0.97	0.91	0.96	0.97	0.95	0.97	x	x	−
4724/03	0.96	**1.05**	**1.04**	**1.11**	**1.07**	1.00	0.94	0.92	0.90	0.90	0.96	0.89	0.93	0.98	0.97	0.96	0.98	0.96	0.95	**1.06**	x	x	−
368/01	0.98	**1.05**	**1.04**	**1.12**	**1.07**	**1.01**	0.93	0.91	0.91	0.90	0.97	0.90	0.93	0.97	0.97	0.93	0.98	0.96	0.94	**1.06**	x	x	−
H37Rv-SR8a2	0.97	0.96	0.97	0.93	0.92	0.94	0.93	0.91	0.91	0.91	0.97	0.91	0.93	0.97	0.97	0.90	0.96	0.96	0.95	0.97	x	x	−
4709/09	**1.10**	**1.05**	**1.04**	**1.11**	**1.06**	**0.99**	0.94	0.93	0.92	0.90	0.97	0.90	0.93	0.98	0.97	0.93	0.98	0.98	0.95	**1.05**	x	x	−
12401/03	0.97	**1.14**	**1.05**	**1.05**	**1.05**	**1.01**	0.93	0.92	0.91	0.90	0.97	0.89	0.93	0.96	0.97	0.95	0.98	0.97	0.95	**1.07**	x	x	−
3355/02	0.97	**1.06**	**1.13**	**1.03**	**1.05**	**1.00**	0.92	0.91	0.90	0.90	0.97	0.89	0.93	0.96	0.97	0.95	0.98	0.96	0.95	**1.06**	x	x	−
3429/03	0.97	0.98	0.97	0.93	0.94	**1.10**	0.87	0.85	**1.07**	0.90	0.97	0.90	0.93	0.96	0.97	0.95	0.98	0.96	0.95	**1.04**	x	x	−
1429/02	0.96	**1.07**	**1.05**	**1.05**	**1.19**	**1.03**	0.93	0.91	0.90	0.90	0.96	0.88	0.93	0.96	0.97	0.95	0.98	0.97	0.94	**1.08**	x	x	−
8085/03	0.97	**1.05**	**1.03**	**1.11**	**1.06**	1.00	**1.06**	0.99	0.96	0.90	0.97	0.88	0.93	0.97	0.97	0.93	0.98	0.96	0.94	**1.09**	x	x	−
853/07	0.97	**1.05**	**1.03**	**1.11**	**1.06**	1.00	0.94	0.92	0.89	0.86	**1.16**	0.97	0.91	0.96	0.96	0.93	0.97	0.96	0.94	**1.08**	x	x	−
H37Rv-SO4a	0.96	0.96	0.96	0.92	0.91	0.94	0.93	0.91	0.91	0.91	0.97	**1.07**	0.93	0.93	0.94	0.90	0.93	0.93	0.93	0.97	x	x	−
4535/04	0.97	**1.06**	**1.04**	**1.11**	**1.07**	**1.00**	0.93	0.92	0.91	0.90	0.98	0.96	**1.08**	***	0.94	1.00	0.95	0.96	***	**1.04**	x	x	−
464/11	0.97	**1.05**	**1.04**	**1.12**	**1.07**	**1.00**	0.93	0.92	0.93	0.90	0.97	0.89	0.93	**1.10**	**1.00**	**1.02**	**1.05**	**1.02**	**1.01**	**1.06**	x	x	−
1598/06	0.97	**1.06**	**1.04**	**1.13**	**1.07**	**1.00**	0.93	0.93	0.91	0.90	0.98	0.90	0.93	**1.03**	**1.00**	**1.04**	**1.02**	**1.00**	**1.03**	**1.06**	x	x	−
10523/05	0.92	**1.05**	**1.04**	**1.11**	**1.06**	**1.00**	0.93	0.91	0.89	0.89	0.98	0.89	0.92	**1.02**	0.98	**1.03**	**1.01**	**1.04**	0.96	**1.07**	x	x	−
3075/05	0.97	**1.14**	**1.05**	**1.04**	**1.05**	**1.00**	0.92	0.91	0.90	0.89	0.97	0.89	0.91	**1.05**	**1.10**	**1.05**	**1.06**	**1.03**	0.99	**1.08**	x	x	−
8444/05	0.95	0.96	0.97	0.92	0.92	0.94	0.88	0.87	**1.06**	0.90	0.97	0.92	0.92	0.97	**1.04**	0.93	**1.12**	**1.04**	0.98	0.95	x	x	−

a Nonboldface numbers indicate detection of the wild-type genotype, and boldface numbers indicate detection of the mutant genotype. The highest discrimination factor is underlined. *, no reaction of the mutant probe (strong discrimination); **, cross-reaction with mutant 533Pro (probe overlapping) with no clear reaction or a weak reaction; ***, clear identification in raw data; x, positive reaction of the assay controls; −, negative reaction.

### Clinical isolates.

To evaluate the clinical value of our melting curve assay, 265 isolates of different MTBC strains from Swaziland were investigated (Table 2). One hundred forty-four of the isolates tested showed a wild-type genotype within the *rpoB* RRDR as well as in codon 572. All these isolates could be correctly identified. The other 121 isolates carried mutations in these targets determining rifampin resistance. Rifampin resistance-mediating mutations could be identified in 99.2% (120/121) of the samples. One isolate exhibiting a *rpoB* deletion (codons 513 and 514) was misclassified by our assay as a mutant carrying a Ser513Leu change [C(A/T)A]. For three isolates with linked mutations, i.e., Asp516Phe [(G/T)(A/C)C] and Asn518Asp [(A/G)AC], we could detect the variant in codon 516 and observed a missing mutant probe for codon 518. The most common mutations in our strain collection were Ser531Leu [T(C/T)G] (44.63% of isolates) and Ile572Phe [(A/T)TC] (33.06%). Additionally, the following mutations were detected: His526Asp [(C/G)AC; 4.96% of isolates], His526Leu [C(A/T)C; 4.96%], His526Tyr [(C/T)AC; 4.96%], Asp516Phe [(G/T)(A/C)C; 3.31%], Asp516Tyr [(G/T)AC; 0.83%], Asp516Val [G(A/T)C; 0.83%], and Ser531Trp [T(C/G)G; 0.83%].

**TABLE 2:** Clinical evaluation of M. tuberculosis isolates from Swaziland

Assay	No. (%) of isolates^*a*^						
	RIF		INH		FQ		Total
	Wild type	Mutant	Wild type	Mutant	Wild type	Mutant^*b*^	
Melting curve assay	144 (54.3)	121 (45.7)	143 (54.0)	122 (46.0)	259 (97.7)	6 (2.3)	265 (100)
Sequencing	144 (54.3)	121 (45.7)	142 (53.6)	123 (46.4)	259 (97.7)	6 (2.3)	265 (100)

a RIF, rifampin; INH, isoniazid; FQ, fluoroquinolones.

b Excluding the phylogenetic SNP Ser95Thr.

Isoniazid resistance-associated mutations in the target region of *katG* and in the *inhA* promoter region were identified in 99.2% (122/123) of all isolates. One isolate with a rare mutation in *katG* (Met275Ile) that is not interrogated by our assay was not identified. The mutations detected were the *katG* mutations Ser315Thr [A(G/C)C; 91.06% and Ser315Gly (A/G)GC; 4.07%] and the −15C → T (26.83%) mutation in the *inhA* promoter region.

The gene *gyrA* is the major target for mutations mediating resistance to fluoroquinolones. Six of the investigated isolates showed such mutations: Asp94Gly [G(A/G)C; 50.00% of isolates], Asp94Tyr (33.33%), and Asp94Asn [(G/A)AC; 16.67%]. All isolates were correctly identified. The phylogenetic SNP in *gyrA* Ser95Thr [A(G/C)C, which is specific for clinical isolates phylogenetically related to the H37Rv reference strain] was correctly identified in 242/242 (100%) of the isolates.

Overall, our assay detected 963/968 (99.5%) of the known resistance targets (four gene regions in 265 isolates) as the correct wild-type or mutant sequence, as previously confirmed by Sanger sequencing.

### Assay performance using crude culture material.

The potential of our melting curve assay for use in a nonlaboratory environment was characterized by the investigation of crude culture material. For this purpose, four different MTBC strains carrying six different mutations in the *rpoB*, *katG*, and *gyrA* genes and in the *inhA* promoter region were selected. Crude cell culture extracts as well as the genomic DNA of these strains were tested, and the results obtained by the melting curve assay were compared. All mutations could be clearly identified, and no difference in the performance of the assay with genomic DNA and the performance of the assay with crude culture material with regard to the calculated discrimination factors was observed (Table S2).

## DISCUSSION

The melting curve assay utilizing the Alere q analyzer platform can be performed with the DNA from patient-derived (subcultured) MTBC isolates as well as with crude cell material. The test is able to rapidly and reliably detect clinically relevant mutations that mediate resistance to three major anti-TB drugs, i.e., rifampin, isoniazid, and fluoroquinolones.

Rifampin resistance is mainly caused by mutations in the *rpoB* RRDR and in *rpoB* codon 572 (8–13). Until now, more than 50 mutations have been identified within the *rpoB* RRDR. Mutations in codons 526 and 531 are well-known to be markers for high-level resistance to rifampin, and their detection is therefore of high importance in predicting the resistance profile (51–53). In addition, the molecular detection of the mutations Leu533Pro [C(T/C)G], Leu511Pro [C(T/C)G], Asp516Tyr [(G/T)AC], and His526Leu [C(A/T)C] have also been described to be linked to treatment failures observed by the use of standard regimens with 600 mg/day rifampin (54, 55). Individual RRDR mutations can confer either low- or high-level resistance, depending on the amino acid change (56). The possibility that high-dose rifampin treatment or treatment with rifabutin instead of rifampin could be used to overcome the resistance conferred by individual RRDR mutations has been discussed, although such treatments for this purpose remain controversial. This highlights the importance of differentiating between the various resistance markers to determine more individualized treatment options that could improve treatment outcomes (57, 58). Furthermore, we implemented the corresponding probes for the detection of the mutation Phe572Ile [(A/T)TC] due to its ability to confer resistance to rifampin, as shown in recent studies (11–13).

Studies have also shown that resistance to isoniazid is very common in countries with a high burden of TB and that isolates resistant to isoniazid may not necessarily be coresistant to rifampin (53). Thus, there is a need to test MTBC strains for isoniazid resistance (59, 60). To obtain proof of isoniazid resistance, we applied probes representing the sequences of the wild type and mutant variants with mutations in *katG* codon 315 as well as at positions −15 and −8 in the *inhA* promoter region. Specific mutations in the *katG* gene are associated with high-level isoniazid resistance, whereas mutations in the *inhA* promoter region, especially the −15C → T mutation, are the cause of high-level resistance to ethionamide and prothionamide and low-level resistance to isoniazid (61). Thus, the −15C → T mutation could also lead to the exclusion of ethionamide and prothionamide from MDR-TB treatment regimens.

Resistance to fluoroquinolones is mainly caused by mutations in the *gyrA* gene (10, 14, 15). The mutations Ala90Val [G(C/T)G], Asp94Asn [(G/A)AC], and Asp94Val [G(A/T)C] were observed to be related to high-level resistance (62, 63). In addition, several mutations in the *gyrB* gene have been detected; however, these have not been frequently detected and are usually detected in association with *gyrA* mutations (9, 47, 64–67). Fluoroquinolones play an important role in the treatment of MDR-TB and are widely used for the treatment of MDR-TB due to the relatively low incidence of side effects in association with their use and their high bactericidal activity compared to the activities of other anti-TB drugs (10). Currently, new fluoroquinolones are being evaluated in clinical trials in an effort to shorten the long-term fluoroquinolone treatments currently used and the associated burden for TB patients (68, 69). All in all, our assay can be used for the detection of the most important mutations which are associated with resistance to rifampin and isoniazid as well as to fluoroquinolones. The results of this assay could possibly help to determine more effective treatment options as a result of determination of the mutations present in isolates from individual patients. The prototype cartridge was designed with a focus on MDR-TB detection and cannot detect resistance to second-line injectable drugs; however, it will be easy to adjust the assay by adjusting the respective probes on the array. The same is true in the event that the focus swings to other mutations or drugs, as the maximum probe number has not been achieved, and an extension of the analysis spectrum is possible.

Overall, we tested 30 genomic DNAs from different MTBC strains for the initial evaluation of the performance of the melting curve assay. With one exception, the mutations in all isolates carrying mutations in one of the respective target regions were correctly identified. Isolate 853/07, which has the uncommon mutation Leu511Arg [C(T/G)G], could not be identified as the corresponding probe for that mutation was not applied on the array. Furthermore, the *rpoB* mutation Ile569Val [(A/G)TC], which occurs outside the *rpoB* RRDR (isolate 10523/05) and which is likewise a rare mutation and in most cases is connected to mutations in the *rpoB* RRDR, was also not detected (70).

To test the performance of the assay in settings with a high incidence of MDR-TB, we tested 265 isolates from a recent drug resistance survey in Swaziland, a country with one of the highest burdens of HIV infection and TB globally (1). Among 116 multidrug-resistant strains included in the same drug resistance survey from 2009 and 2010, Sanchez-Padilla et al. observed 38 (32.8%) strains that carried the *rpoB* Ile572Phe [(A/T)TC] mutation, which confers resistance to rifampin (23, 71). Although this mutation was previously reported at a low frequency in clinical isolates in Hong Kong and Australia (72), we decided to implement the detection of the *rpoB* Ile572Phe [(A/T)TC] mutation in our melting curve assay. Overall, the resistance-mediating mutations previously identified by Sanger sequencing were confirmed by our assay to be present in all fluoroquinolone-resistant isolates and in 99.2% of the isoniazid- and rifampin-resistant isolates. The few exceptions were the mutation consisting of the deletion of codons 513 and 514 in *rpoB* in one isolate that was misinterpreted as having the Ser513Leu [C(A/T)A] mutation. Three isolates carrying linked mutations in the *rpoB* RRDR in codons 516 and 518 were identified to have the correct mutation in codon 516, but for codon 518 we misinterpreted the wild-type sequence due to the absence of an Asn518Asp [(A/G)AC] probe in our assay. The mutation *rpoB* Ala633Cys [(G/T)(C/G)C] of isolate 4723/09 is not associated with resistance. In this case, rifampin resistance was correctly predicted by detection of the Ile572Phe [(A/T)TC] mutation in *rpoB*. The rare proposed isoniazid resistance marker *katG* Asn275Ile [A(C/T)C] could not be identified in isolate 1057/10 because this mutation is, like the *rpoB* mutation in codon 633, located outside the defined areas where mutations are detected by the assay. Moreover, a clear association between these rare mutations and their impact on the resistance phenotype is still missing (73, 74). These examples of rare and partially unknown mutations also show the conceptual limitations of target-based molecular tests in general. The predefined set of clinically and epidemiologically relevant mutations will need to be carefully selected and is limited, and the evaluation of particular mutations might differ even in adjacent geographic regions, as observed in KwaZulu-Natal in South Africa and Swaziland, which have distinct MDR/XDR strain types and where the strains have distinct mutation profiles (71, 75).

The gold standard for the detection of drug resistance is still the culture-based proportion method with Lowenstein-Jensen medium, which is recommended by WHO and has been used for over 50 years (76). However, due to a generation time of MTBC strains of approximately 24 h, culture-based methods are very slow and technically challenging and require a well-established laboratory infrastructure. Consequently, these aspects delay the detection of drug resistance and lead to inappropriate treatment and the spread of drug-resistant strains (77). Fast culture methods, such as those that use the Bactec MGIT 960/BactT/Alert system and the phage technique, are also tedious and require a biosafety level 3 laboratory environment and specialized staff (78, 79). Therefore, and because drug resistance is associated with mutations which code for resistance to the respective drug target or for enzymes involved in the drug inactivation process, molecular test systems are now widely used in (supra-)national TB reference laboratories (80). Several commercial assays that test for rifampin, isoniazid, and fluoroquinolone resistance are available. The results of line probe assays, such as the GenoType MTBDRplus assay (v1.0 and v2.0) and the GenoType MTBDRsl assay (v2.0), which detect mutations mediating resistance to rifampin, isoniazid, fluoroquinolones, and ethambutol, as well as to injectable drugs, have shown a high concordance with those of standard diagnostic methods (culture, real-time PCR) and DNA sequencing (81, 82). Despite its high sensitivity and specificity, the open platform design harbors a risk for potential amplicon contamination, and its performance is still labor-intensive (83). The Xpert MTB/RIF assay is the first point-of-care system that enables the sensitive and specific detection of rifampin resistance by the direct use of primary sputum samples. In summary, after preparation of the sputum specimen, all subsequent steps, including PCR amplification and interpretation of the results, are fully automated (84, 85). So far, the Xpert system is limited to the detection of rifampin resistance, and other proposed molecular test systems are also suitable for the detection of multidrug resistance, but they require several manual handling steps and specialized equipment (86–88).

In conclusion, our melting curve assay with a prototype cartridge is suitable for the detection of rifampin, isoniazid, and fluoroquinolone resistance, and from the time of application of the reaction mix to the time of detection, the assay can be completed within 90 min. The assay reliably detects MTBC DNA in amounts down to 24 copies per reaction mixture and works with genomic DNA as well as with crude cell extracts. The prototype cartridge has the advantage that it can be run with dried reagents, which has been confirmed with wild-type genomic DNA (see Fig. S1 in the supplemental material). Furthermore, the array system is very flexible, and resistance markers as well as the maximum amount of spotted probes can be adapted to future diagnostic requirements in a straightforward manner.

## Supplementary Material

Supplemental material
