# Supplementary material for: Rapid Microarray-Based Detection of Rifampin, Isoniazid, and Fluoroquinolone Resistance in Mycobacterium tuberculosis by Use of a Single Cartridge
Source: J Clin Microbiol. 2018 Jan 24;56(2):e01249-17. doi: 10.1128/JCM.01249-17 (PMC5786735; doi:10.1128/JCM.01249-17)
Supplement: Supplemental material [file supp_56_2_e01249-17__index.html]

Supplemental material 

# Rapid Microarray-Based Detection of Rifampin, Isoniazid, and Fluoroquinolone Resistance in Mycobacterium tuberculosis by Use of a Single Cartridge

## Supplemental material

- Supplemental file 1 -

  Fig. S1 (Comparison of liquid and dried reagents)

  PDF, 709K
- Supplemental file 2 -

  Table S1 (Analyzed *M. tuberculosis* isolates)

  PDF, 261K
- Supplemental file 3 -

  Table S2 (Results of genomic DNA and crude culture extracts in the melting curve assay)

  PDF, 311K
- Supplemental file 4 -

  Table S3 (Primers, probe variants, and TaqMan probes)

  PDF, 532K
