# Supplementary material for: Rapid Microarray-Based Detection of Rifampin, Isoniazid, and Fluoroquinolone Resistance in Mycobacterium tuberculosis by Use of a Single Cartridge
Source: J Clin Microbiol. 2018 Jan 24;56(2):e01249-17. doi: 10.1128/JCM.01249-17 (PMC5786735; doi:10.1128/JCM.01249-17)
Supplement: Supplemental material [file JCM.01249-17_zjm999095824s1.pdf]

Fig. S1. Comparison of liquid and dried reagents

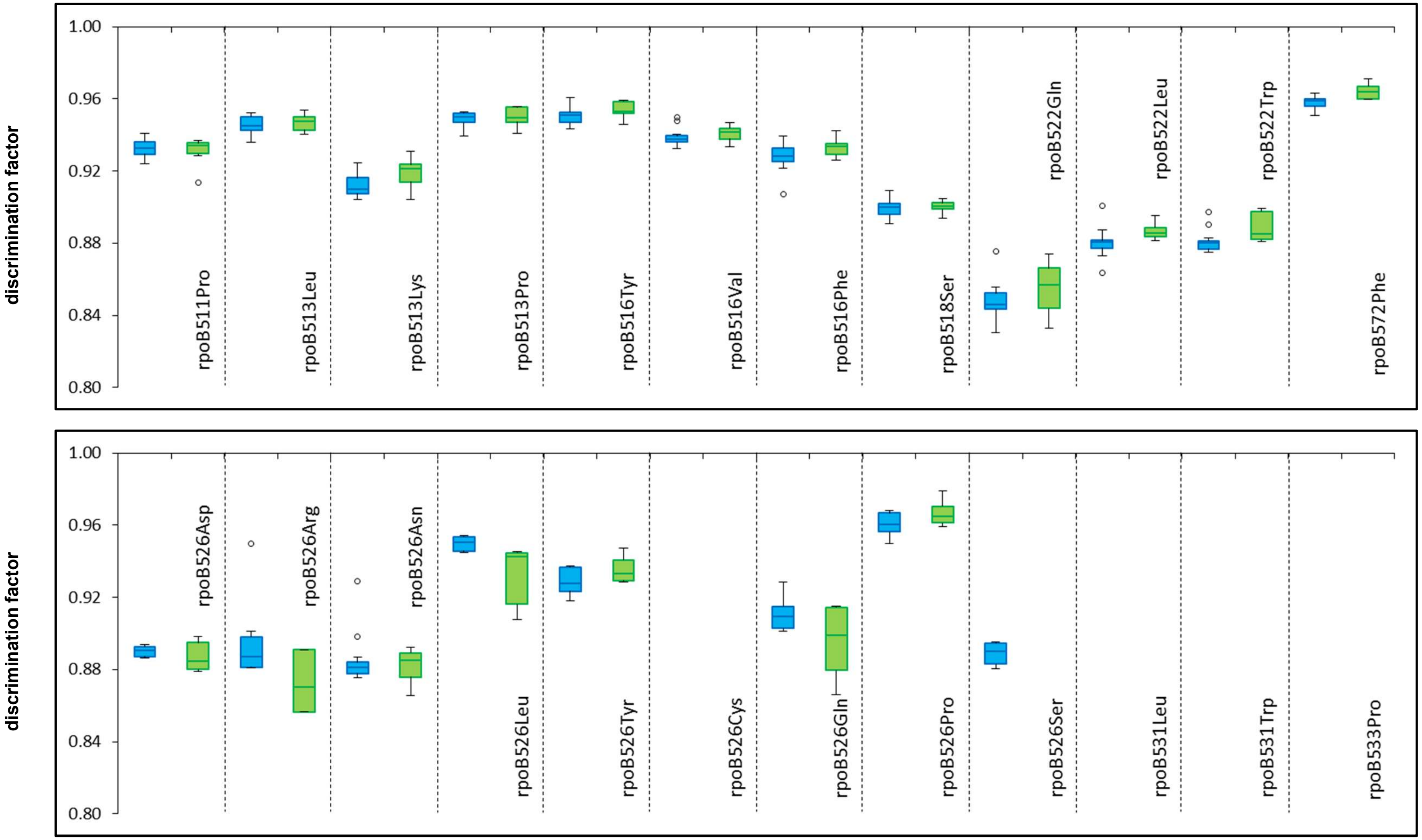

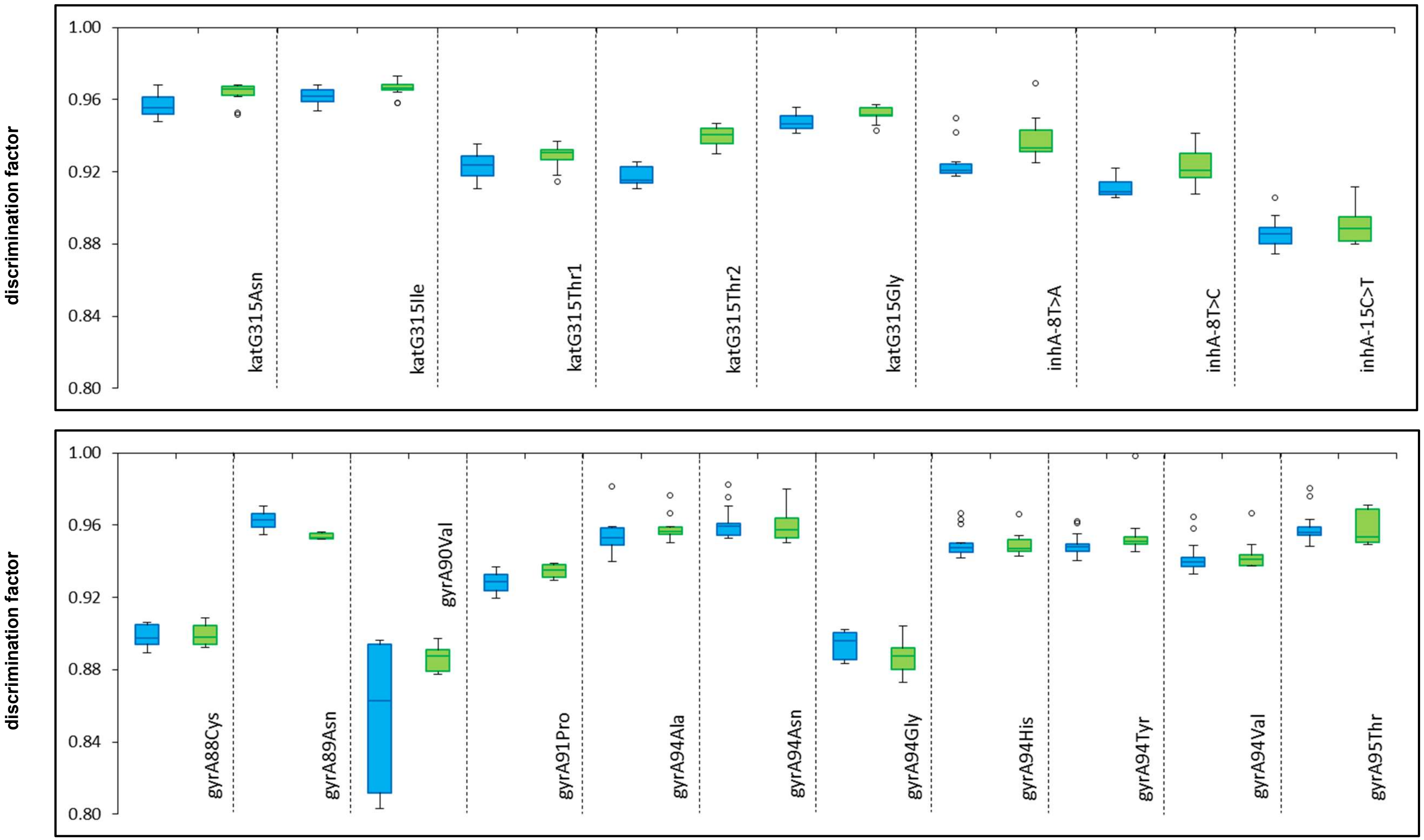

Liquid (blue) and dried (green) reagents in the amplification mix were used in the Alere^{TM} q cartridge for the melting curve assay. The analysis was performed with 500 copies per reaction of the *M. tuberculosis* reference strain H37Rv. The discrimination factors are given for all targets. Due to the wild type genotype a discrimination factor < 1 was determined on all

mutant probes. In some cases the discrimination was so strong that a valid discrimination factor was only determined for the wild type probes and none for the mutant probes. The circles indicate an outlier of the corresponding data series.
