# Supplementary material for: Rapid Microarray-Based Detection of Rifampin, Isoniazid, and Fluoroquinolone Resistance in Mycobacterium tuberculosis by Use of a Single Cartridge
Source: J Clin Microbiol. 2018 Jan 24;56(2):e01249-17. doi: 10.1128/JCM.01249-17 (PMC5786735; doi:10.1128/JCM.01249-17)
Supplement: Supplemental material [file JCM.01249-17_zjm999095824s2.pdf]

Table S1. Analyzed *M. tuberculosis* isolates

Isolate	Genotype				
	rpoB hs	*rpoB* 572	*katG*	*inhA*	*gyrA*
H37Rv	wild type	wild type	wild type	wild type	wild type
3736/04	Leu511Pro: C(T/C)G	wild type	wild type	wild type	Ser95Thr: A(G/C)C
698/05	Ser513Pro: C(A/C)A	wild type	Ser315Thr: A(G/C)C	wild type	Ser95Thr: A(G/C)C
10502/06	Ser513Leu: C(A/T)A	wild type	Ser315Thr: A(G/C)C	wild type	Ser95Thr: A(G/C)C
6695/04	Asn518Ser: A(A/G)C<br>Leu533Pro: C(T/C)G	wild type	Ser315Thr: A(G/C)C	wild type	Ser95Thr: A(G/C)C
7941/01	Ser522Leu: T(C/T)G	wild type	wild type	wild type	Ser95Thr: A(G/C)C
49-02-SR4a	Ser522Trp: T(C/G)G	wild type	wild type	wild type	Ser95Thr: A(G/C)C
5976/01	His526Leu: C(A/T)C	wild type	Ser315Thr: A(G/C)C	wild type	Ser95Thr: A(G/C)C
4787/03	His526Tyr: (C/T)AC	wild type	Ser315Thr: A(G/C)C	wild type	Ser95Thr: A(G/C)C
3307/03	His526Asn: (C/A)AC	wild type	wild type	-15 C→T	Ser95Thr: A(G/C)C
10427/01	His526Cys: (C/T)(A/G)C	wild type	Ser315Thr: A(G/C)C	wild type	Ser95Thr: A(G/C)C
2822/06	His526Asp: (C/G)AC	wild type	wild type	-15 C→T	Ser95Thr: A(G/C)C
H37Rv-SR4k	His526Pro: C(A/C)C	wild type	wild type	wild type	wild type
4724/03	His526Arg: C(A/G)C	wild type	Ser315Thr: A(G/C)C	wild type	Ser95Thr: A(G/C)C
368/01	Ser531Leu: T(C/T)G	wild type	Ser315Thr: A(G/C)C	wild type	Ser95Thr: A(G/C)C
H37Rv-SR8a2	Ser531Trp: T(C/G)G	wild type	wild type	wild type	wild type

Isolate	Genotype				
	rpoB hs	*rpoB* 572	*katG*	*inhA*	*gyrA*
4709/09	wild type	Ile572Phe: (A/T)TC	Ser315Thr: A(G/C)C	wild type	Ser95Thr: A(G/C)C
12401/03	Ser531Leu: T(C/T)G	wild type	Ser315Asn: A(G/A)C	wild type	Ser95Thr: A(G/C)C
3355/02	wild type	wild type	Ser315Ile: A(G/T)C	wild type	Ser95Thr: A(G/C)C
3429/03	wild type	wild type	Ser315Gly: (A/G)GC	-15 C→T	Ser95Thr: A(G/C)C
1429/02	wild type	wild type	Ser315Thr: A(G/C)(C/A)	wild type	Ser95Thr: A(G/C)C
8085/03	Asp516Val: G(A/T)C	wild type	Ser315Thr: A(G/C)C	-8 T→A	Ser95Thr: A(G/C)C
853/07	Leu511Arg: C(T/G)G<br>Asp516Tyr: (G/T)AC	wild type	Ser315Thr: A(G/C)C	wild type	Asp89Asn: (G/A)AC<br>Ser95Thr: A(G/C)C
H37Rv-SO4a	wild type	wild type	wild type	wild type	Ala90Val: G(C/T)G
4535/04	Ser531Leu: T(C/T)G	wild type	Ser315Thr: A(G/C)C	wild type	Ser91Pro: (T/C)CG<br>Ser95Thr: A(G/C)C
464/11	Ser531Leu: T(C/T)G	wild type	Ser315Thr: A(G/C)C	wild type	Asp94Ala: G(A/C)C<br>Ser95Thr: A(G/C)C
1598/06	Ser531Leu: T(C/T)G	wild type	Ser315Thr: A(G/C)C	wild type	Asp94Gly: G(A/G)C<br>Ser95Thr: A(G/C)C
10523/05	Ser531Leu: T(C/T)G	Ile569Val: (A/G)TC	Ser315Thr: A(G/C)C	wild type	Asp94Tyr: (G/T)AC<br>Ser95Thr: A(G/C)C

Isolate	Genotype				
	rpoB hs	*rpoB* 572	*katG*	*inhA*	*gyrA*
3075/05	Ser531Leu: T(C/T)G	wild type	Ser315Asn: A(G/A)C	wild type	Asp94Asn: (G/A)AC<br>Ser95Thr: A(G/C)C
8444/05	Ser531Leu: T(C/T)G	wild type	wild type	-15 C→T	Asp94His: (G/C)AC
