# Supplementary material for: Rapid Microarray-Based Detection of Rifampin, Isoniazid, and Fluoroquinolone Resistance in Mycobacterium tuberculosis by Use of a Single Cartridge
Source: J Clin Microbiol. 2018 Jan 24;56(2):e01249-17. doi: 10.1128/JCM.01249-17 (PMC5786735; doi:10.1128/JCM.01249-17)
Supplement: Supplemental material [file JCM.01249-17_zjm999095824s3.pdf]

Table S2. Results of genomic DNA and crude culture extracts in the melting curve assay

Samples \ Probes	*rpoB* RRDR																						
	511Pro_v02	513Leu_v03	513Lys_v02	513Pro_v04	516Tyr_v03	516Val_v03	516Phe_v03	518Ser_v02	522Gln_v03	522Leu_v02	522Trp_v01	526Asp_v04	526Arg_v04	526Asn_v06	526Leu_v04	526Tyr_v04	526Cys_v03	526Gln_v03	526Pro_v04	526Ser_v01	531Leu_v04	531Trp_v05	533Pro_v04
**g4711/09**	0.94	0.94	0.92	0.96	0.96	0.96	0.96	0.92	0.88	0.92	0.90	0.89	0.87	0.91	0.92	0.91	0.86	0.89	0.93	0.86	0.92	0.90	0.90
**c4711/09**	0.94	0.95	0.92	0.96	0.96	0.95	0.95	0.92	0.89	0.93	0.91	0.89	0.87	0.91	0.92	0.91	0.86	0.89	0.93	0.87	0.93	0.90	0.89
**g10114/09**	0.94	0.95	0.92	0.96	0.95	0.95	0.95	0.91	0.87	0.90	0.89	**1.19**	0.96	1.07	0.97	1.06	0.98	0.92	0.96	0.98	0.91	0.89	0.91
**c10114/09**	0.95	0.95	0.92	0.96	0.96	0.95	0.95	0.91	0.88	0.89	0.90	**1.18**	0.96	1.07	0.97	1.06	0.98	0.92	0.96	0.98	0.92	0.90	0.92
**g5772/09**	0.94	0.94	0.91	0.95	0.95	0.95	0.95	0.91	0.85	0.91	0.88	0.89	0.87	0.90	0.92	0.91	0.86	0.89	0.94	0.87	**1.10**	0.97	*
**c5772/09**	0.94	0.93	0.89	0.94	0.95	0.94	0.94	0.90	0.84	0.87	0.87	0.88	0.89	0.89	0.93	0.91	0.86	0.90	0.94	0.88	**1.08**	0.96	*
**g10298/09**	0.94	0.94	0.92	0.96	0.95	0.95	0.95	0.92	0.87	0.92	0.90	0.91	0.89	0.91	0.94	0.93	0.87	0.91	0.95	0.88	0.94	0.92	*
**c10298/09**	0.95	0.95	0.92	0.96	0.96	0.96	0.95	0.92	0.89	0.93	0.90	0.90	0.88	0.91	0.93	0.92	0.87	0.90	0.95	0.88	0.94	0.91	*

Samples \ Probes	*rpoB*	*katG*					*inhA*			*gyrA* QRDR											Controls		
	572Phe_v04	315Asn_v06	315Ile_v01	315Thr1_v01	315Thr2_v04	315Gly_v04	-8T>A_v02	-8T>C_v02	-15C>T_v02	88Cys_v01	89Asn_v04	90Val_v01	91Pro_v04	94Ala_v01	94Asn_v02	94Gly_v02	94His_v04	94Tyr_v02	94Val_v02	95Thr_v01	Msme	MTB	sp_02
**g4711/09**	0.94	0.96	0.95	0.91	0.89	0.95	0.92	0.91	0.89	0.89	0.96	0.87	0.92	0.93	0.93	0.86	0.93	0.93	0.92	0.94	x	x	-
**c4711/09**	0.94	0.97	0.95	0.92	0.90	0.95	0.92	0.91	0.89	0.89	0.96	0.88	0.91	0.93	0.93	0.86	0.93	0.93	0.93	***	x	x	-
**g10114/09**	0.95	1.07	1.03	**1.11**	1.05	1.01	0.93	0.91	0.88	0.90	0.96	0.87	0.92	1.03	0.98	1.07	1.03	1.00	1.05	**1.10**	x	x	-
**c10114/09**	0.96	1.08	1.04	**1.12**	1.05	1.01	0.92	0.91	0.87	0.90	0.97	0.88	0.90	1.03	0.99	1.06	1.02	1.00	1.04	**1.10**	x	x	-
**g5772/09**	0.95	1.09	1.04	**1.12**	1.06	1.02	0.88	0.86	**1.08**	0.90	0.97	0.88	0.92	0.95	0.94	0.90	0.96	0.94	0.91	**1.09**	x	x	-
**c5772/09**	0.96	1.08	1.05	**1.13**	1.05	1.02	0.91	0.92	**1.07**	0.89	0.97	0.90	0.93	0.96	0.96	0.94	0.97	0.95	0.94	**1.08**	x	x	-
**g10298/09**	**1.10**	1.07	1.04	**1.11**	1.05	1.01	0.92	0.91	0.88	0.89	0.97	0.88	0.91	0.96	0.95	0.93	0.97	0.95	0.93	**1.09**	x	x	-
**c10298/09**	**1.09**	1.07	1.03	**1.11**	1.05	1.01	0.93	0.91	0.88	0.90	0.96	0.87	0.91	0.96	0.95	0.93	0.96	0.94	0.92	**1.09**	x	x	-

For each genomic DNA (g) and its corresponding crude culture extract (c) the determined discrimination factor (n = 2 to 3) is given for the different mutant probes in the respective target regions. Blue color represents a wild type and yellow color a mutant detection. The results of the assay controls are shown in the last three columns. The *M. tuberculosis* specific positive control “MTB” was positive in the presence of *M. tuberculosis* DNA. The internal process control “Msme” was positive and the hybridization control “sp02” was negative in all reactions.
