# Supplementary material for: Rapid Microarray-Based Detection of Rifampin, Isoniazid, and Fluoroquinolone Resistance in Mycobacterium tuberculosis by Use of a Single Cartridge
Source: J Clin Microbiol. 2018 Jan 24;56(2):e01249-17. doi: 10.1128/JCM.01249-17 (PMC5786735; doi:10.1128/JCM.01249-17)
Supplement: Supplemental material [file JCM.01249-17_zjm999095824s4.pdf]

Table S3. Primers, probe variants and TaqMan® probes

Primers

Target	Primer	Sequence (5´-3´)	Product size (bp)	Sequence length (bp)	T_m (°C)
rpoB[a] *RRDR*	*rpoB* hs fw	CCGCGATCAAGGAGTTCTTCG	129	21	58.7
	rpoB hs rv	CACGCTCACGTGACAGACC		19	58.4
rpoB[a] *572*	*rpoB* 572 fw	GTCGCACTACGGCCGGA	158	17	59.3
	rpoB 572 rv	ACACGATCTCGTCGCTAACCA		21	58.9
katG[b]	*katG* fw	GCTGGAAGAGCTCGTATGGC	146	20	58.2
	katG rv	AGGGCTCTTCGTCAGCTCC		19	58.7
inhA[c]	*inhA* fw	CGGAAATCGCAGCCACGT	130	18	58.5
	inhA rv	GGACTGAACGGGATACGAATGG		22	58.1
gyrA[d] QRDR	*gyrA* fw	CGCAGCCACGCCAAGTC	134	17	59.3
	gyrA rv	CCGTCCACCAGCGGGTAG		18	59.5
process control[e]	Msme fw	GGTGGACGACGAGGAGCTCA	105	20	61.0
	Msme rv	CCGAGACGCGGACCACG		17	60.4

[a] Rv0667
[b] Rv1908c
[c] Rv1484
[d] Rv0006
[e] MSMEG_1401

Probe variants

Drug	Locus	Codon	Mutation	Names	Sequence (5´-3´)
Rifampicin	*rpoB*	511	wild type	amino Leu511wt	GAATTGGCTCAGCTGGCTGGTGC
			Leu511Pro	rpoB_511pro_v02	CATGAATTGGCTCGGCTGGCTGG
		513	wild type	rpoB_513wt_v03	TCTGGTCCATGAATTGGCTCAGCTGG
			Ser513Leu	rpoB_513Leu_v03	GTTCTGGTCCATGAATAGGCTCAGCTGG
			Ser513Lys	rpoB_513Lys_v02	TGGTCCATGAATTTGCTCAGCTGGCT
			Ser513Pro	rpoB_513Pro_v04	GGTCCATGAATGGGCTCAGCTGGCTG
		516	wild type	rpoB_516wt_v01	GGGTTGTTCTGGTCCATGAATTGGCTCA
			Asp516Phe	rpoB_516Phe_v03	GCGGGTTGTTCTGGAACATGAATTGGC
			Asp516Tyr	rpoB_516Tyr_v03	AGCGGGTTGTTCTGGTACATGAATTGGC
			Asp516Val	rpoB_516Val_v03	AGCGGGTTGTTCTGGACCATGAATTGG
		518	wild type	rpoB_518wt_v02	GACAGCGGGTTGTTCTGGTCCATGA
			Asn518Ser	rpoB_518Ser_v02	ACAGCGGGTTGCTCTGGTCCATG
		522	wild type	rpoB_522wt_v02	GGTCAACCCCGACAGCGGGT
			Ser522Gln	rpoB_522Gln_v03	TGGGTCAACCCCTGCAGCGG
			Ser522Leu	rpoB_522Leu_v02	GGGTCAACCCCAACAGCGGGT
			Ser522Trp	rpoB_522Trp_v01	GTCAACCCCCACAGCGGGTTGT
		526	wild type	rpoB_526wt_v04	CGGCGCTTGTGGGTCAACCCC
			His526Asn	rpoB_526Asn_v06	GCGCTTGTTGGTCAACCCCGAC
			His526Asp	rpoB_526Asp_v04	AGTCGGCGCTTGTCGGTCAACC
			His526Arg	rpoB_526Arg_v04	CGGCGCTTGCGGGTCAACCC
			His526Cys	rpoB_526Cys_v03	CAGTCGGCGCTTGCAGGTCAAC
			His526Gln	rpoB_526Gln_v03	GTCGGCGCTTTTGGGTCAACCC
			His526Leu	rpoB_526Leu_v04	TCGGCGCTTGAGGGTCAACCCC
			His526Pro	rpoB_526Pro_v04	GTCGGCGCTTGGGGGTCAACCC

Drug	Locus	Codon	Mutation	Names	Sequence (5´-3´)
			His526Ser	rpoB_526Ser_v01	CGGCGCTTGCTGGTCAACCCC
			His526Tyr	rpoB_526Tyr_v04	TCGGCGCTTGTAGGTCAACCCCG
		531	wild type	rpoB_531wt_v05	GGCCCCAGCGCCGACAGTCGG
			Ser531Leu	rpoB_531Leu_v04	GCCCCAGCGCCAACAGTCGGCG
			Ser531Trp	rpoB_531Trp_v05	GGCCCCAGCGCCCACAGTCGG
		533	wild type	rpoB_533wt_v04	CGGGCCCCAGCGCCGACAGTC
			Leu533Pro	rpoB_533Pro_v04	CGGGCCCCGGCGCCGACAG
		572	wild type	poB_572wt_v03	CGACAGCGAGCCGATCAGACCG
			Ile572Phe	rpoB_572Phe_v04	CAGCGAGCCGAACAGACCGATGTTGG
Isoniazid	*katG*	315	wild type	katG_315wt_v03	ACCTCGATGCCGCTGGTGATCG
			Ser315Asn	katG_315Asn_v06	ACGACCTCGATGCCGTTGGTGATC
			Ser315Gly	katG_315Gly_v04	ACCTCGATGCCGCCGGTGATCGC
			Ser315Ile	katG_315Ile_v01	CTCGATGCCGATGGTGATCGCGT
			Ser315Thr	katG_315Thr1_v01	CGATGCCGGTGGTGATCGCGT
				katG_315Thr2_v04	CCTCGATGCCTGTGGTGATCGCGT
	inhA	-8	wild type	inhA_08wt_v03	GCAGTCACCCCGACAACCTATCGTCT
			-8T→A	inhA_08T>A_v02	AGTCACCCCGACATCCTATCGTCTCG
			-8T→C	inhA_08T>C_v02	TCACCCCGACAGCCTATCGTCTCG
		-15	wild type	inhA_15wt_v03	CCGACAACCTATCGTCTCGCCGC
			-15C→T	inhA_15C>T_v02	CGACAACCTATCATCTCGCCGCGG
Fluoroquinolones	*gyrA*	88	wild type	gyrA_88wt_v01	GTCGCCGTGCGGGTGGTAGT
			Gly88Cys	gyrA_88Cys_v01	CGTCGCAGTGCGGGTGGTAGT
		89	wild type	gyrA_89wt_v05	GTAGATCGACGCGTCGCCGTGCGG
			Asp89Asn	gyrA_89Asn_v04	GATCGACGCGTTGCCGTGCGGGTG
		90	wild type	gyrA_90wt_v04	CGTAGATCGACGCGTCGCCGTGC

Drug	Locus	Codon	Mutation	Names	Sequence (5´-3´)
			Ala90Val	gyrA_90val_v01	AGATCGACACGTCGCCGTGCG
		91	wild type	gyrA_91wt_v04	TCGTAGATCGACGCGTCGCCGTGCG
			Ser91Pro	gyrA_91Pro_v04	TCGTAGATCGGCGCGTCGCCGTGC
		94	wild type	gyrA_94wt_v03	CGCACCAGGCTGTCGTAGATCGAC
			Asp94Ala	gyrA_94Ala_v01	CCAGGCTGGCGTAGATCGACGC
			Asp94Asn	gyrA_94Asn_v02	GCACCAGGCTGTTGTAGATCGACGC
			Asp94Gly	gyrA_94Gly_v02	GCACCAGGCTGCCGTAGATCGAC
			Asp94His	gyrA_94His_v04	CGCACCAGGCTGTGGTAGATCGACGC
			Asp94Tyr	gyrA_94Tyr_v02	GCACCAGGCTGTAGTAGATCGACGC
			Asp94Val	gyrA_94Val_v02	GCACCAGGCTGACGTAGATCGACG
		95	wild type	gyrA_95wt_v01	CCATGCGCACCAGGCTGTCGTAGATCGAC
			Ser95Thr	gyrA_95Thr_v01	CCATGCGCACCAGGGTGTCGTAGATCGAC
Assay controls				MTB	CCGAAATCGGTATGTCCACGAGCGT
				Msme	GTCCTGGGCGGCCAGCAGTT
				sp02	TCCGCTACTCGGGATCAGGGAGC

TaqMan® probes

TaqMan® probe	Sequence (5´-3´)	5´ Modification	3´ Modification
*rpoB*_531wt	CCAGCGCCGACAGTCGG	FAM	BHQ1
*rpoB*_572wt	CGGTCTGATCGGCTCGCTGTCG	TAMRA	BHQ2
*katG*_315wt	GACCTCGATGCCGCTGGTG	Cy5	BHQ3
*inhA*_-8wt	CACCCCGACAACCTATCGTCTC	ROX	BHQ2
*gyrA*_94wt	CGTCGATCTACGACAGCCTGGTGC	JOE	BHQ1
Msme	GTCCTGGGCGGCCAGCAGTT	TAMRA	BHQ2
